# Behavioural Interventions to Treat Oropharyngeal Dysphagia in Children with Cerebral Palsy: A Systematic Review of Randomised Controlled Trials

**DOI:** 10.3390/jcm14176005

**Published:** 2025-08-25

**Authors:** Michelle McInerney, Sarah Moran, Sophie Molloy, Carol-Anne Murphy, Bríd McAndrew

**Affiliations:** 1 Department of Language & Cognition, Faculty of Brain Sciences, University College London (UCL), London WC1N 1PF, UK; 2 Heart Centre for Children, Westmead Children’s Hospital, Sydney Children’s Hospital Network (SCHN), Sydney, NSW 2145, Australia; 3 Paediatrics & Child Health, School of Clinical Medicine, University of New South Wales (UNSW), Sydney, NSW 2052, Australia; 4 School of Allied Health, University of Limerick, V94 T9PX Limerick, Ireland; 17230918@studentmail.ul.ie (S.M.); 22135367@studentmail.ul.ie (S.M.); carol-anne.murphy@ul.ie (C.-A.M.); 5 Health Research Institute, Centre for Implementation Research, University of Limerick, V94 T9PX Limerick, Ireland; 6 Speech & Language Therapy Department, Children’s Disability Network Team-1, North Mayo, F26 R62W Mayo, Ireland; brid.mcandrew@hse.ie

**Keywords:** cerebral palsy (CP), swallowing disorder, oropharyngeal dysphagia, OPD, behavioural intervention, effectiveness, randomised controlled trials, RCTs, compensatory, skills training

## Abstract

**Background/Objectives**: Swallowing disorder(s), or oropharyngeal dysphagia (OPD), are very common in children with cerebral palsy (CP) and pose a significant risk to their health. Behavioural interventions are frequently recommended when targeting OPD in children with CP; however, their efficacy has yet to be determined. This systematic review aimed to synthesise the current evidence for behavioural interventions in the treatment of OPD in children with CP. **Methods**: A comprehensive search in six databases in October 2024 sought studies that (1) included participants aged 0–18 years with a diagnosis of CP and OPD; (2) utilised and described a behavioural intervention for OPD; and (3) used a randomised controlled trial (RCT) experimental design. Three reviewers independently extracted the data, and results were tabulated. The Revised Cochrane Risk of Bias (ROB-2) tool was used to determine the methodological quality of eligible articles. **Results**: From an initial yield of 2083 papers, 99 full-text studies were screened for eligibility. Seven RCTs involving 329 participants aged 9.5 months (SD = 2.03) to 10.6 yrs were included. CP description varied. Most studies used a combination of behavioural interventions to treat OPD (*n* = 6), and oral sensorimotor treatment was the most frequently utilised treatment (*n* = 4). Positive outcomes were reported in all (*n* = 7); however, there was high risk of bias in five studies. **Conclusions:** The use of behavioural interventions to treat OPD in children with CP continues to be supported by low-level evidence. Rigorously designed RCTs with larger samples of children with CP and OPD are needed to evaluate the true effects of behavioural interventions across the developmental phase of childhood. Importantly, consistency in describing and reporting baseline analysis of swallowing and OPD; together with treatment-component data, is a priority in future research.

## 1. Introduction

Cerebral palsy (CP) is a permanent, lifelong condition which describes a spectrum of motor impairments caused by a non-progressive brain injury during the early developmental period [1]. CP is the most common cause of childhood-onset disability, with a prevalence range of 1.6 per 1000 live births for high-income countries and 3.4 per 1000 live births in low-income countries [2]. The presenting motor impairments, which are core to CP, are frequently accompanied by sensory, perceptual, cognitive, communication, and behavioural difficulties, and secondary musculoskeletal disorders [3,4,5]. Due to a complex interplay of aetiological factors, motoric dysfunction, and associated comorbid features, there is significant heterogeneity in the profiles of children with CP [6].

Oropharyngeal dysphagia (OPD), or swallowing disorder, is a frequently occurring comorbidity in people with CP, with a reported pooled prevalence estimate of 50.4% [7]. Prevalence estimates are higher in children with CP, with reported ranges varying from 60 to 90% [8,9,10]. With a diagnosis of OPD in children with CP, the oral and pharyngeal phases of swallowing are often affected [11]. The oral phase involves two discrete events: Oral preparation, where the food/fluid material is manipulated adequately and formed into a cohesive bolus. Once complete, the tongue elevates to propel the bolus posteriorly until the pharyngeal swallow, and next phase, is initiated [12]. The pharyngeal phase involves directing the bolus into the oesophagus whilst protecting the airway from aspiration [13].

Common features of OPD in children with CP include poor lip closure, tongue thrusting, masticatory inefficiency, choking, and aspiration [14,15]. Gastroesophageal Reflux Disease is also common in CP and can further exacerbate feeding difficulties by causing discomfort and complicating the feeding process [16]. The implications of OPD in CP are often serious, potentially leading to malnutrition and underdevelopment, significant pulmonary compromise, and prolonged stressful mealtimes [17]. Despite high prevalence rates and the evident serious health repercussions for children with CP who present with swallowing impairments, effective treatments to optimise swallowing function and reduce the impact of OPD are lacking [18].

Behavioural interventions are frequently recommended to treat OPD in children with CP [19]. However, previous research highlights conflicting findings regarding treatment effectiveness in the context of poorly designed quasi-experimental research conducted with small samples [20,21]. Traditionally, a host of behavioural interventions including reinforcement, modelling, or shaping can be used by clinicians to modify a desirable or undesirable behaviour [22]. Behavioural interventions for OPD can be further divided into interventions that target compensation and skill training [23]. Compensatory techniques prioritise safety, adapting to or compensating for a deficit in functioning [24], while skill training aims for the individual to acquire new, more advanced skills to increase their independence [25,26], which can lead to long-term change in swallowing. Skill training can be further broken down into direct and indirect training. Direct interventions involve the use of food or fluid items and are goal-oriented task-specific behavioural techniques that optimise skill acquisition, often by harnessing neuroplasticity, e.g., increasing flow rate using faster-flow bottle nipples, and incremental increases to more challenging textures for improved acceptability of foods and improved chewing ability [23,27]. Indirect skill training uses non-nutritive stimuli to increase the resistance and strength of targeted muscles [28,29], e.g., non-nutritive sucking on a pacifier, and oral motor resistance exercises with chewy tubes.

In the current study, the International Classification of Functioning, Disability and Health (ICF) [30] was used as the conceptual framework. The International Classification of Functioning, Disability and Health conceptualises a person’s level of ‘functioning’ as a dynamic interaction between health conditions, environmental factors, and personal factors. Functioning includes two parts related to (1) body functions and structures, which describe the anatomy and physiology/psychology of the human body; and (2) activity and participation, which describe the individuals’ current functional level related to skills like mobility, self-care communication, and learning [31].

Given the paucity of high-quality evidence in relation to interventions for children with CP, this systematic review aimed to determine the effects of behavioural interventions in children with CP based only on the highest level of evidence (randomised controlled trials (RCTs)). Behavioural interventions that targeted a behaviour related to feeding, eating, drinking, and/or swallowing and which were delivered by a professional dysphagia expert were the focus of this review. Dysphagia experts could include the disciplines of speech and language pathologists, occupational therapists, or physiotherapists but could also incorporate other wider discipline groups relevant to this study’s health service/system. Surgical, pharmacological measures and neuromuscular electrical stimulation (NMES) to treat OPD were considered outside the scope of this review.

## 2. Materials and Methods

This systematic review was designed in accordance with the Preferred Reporting Items for Systematic Reviews and Meta-Analyses (PRISMA) [32]. Covidence (Veritas Health Innovation, Melbourne, Australia) was utilised as the primary platform for screening and inclusion of studies, facilitating an efficient and organised review process. 

### 2.1. Information Sources

To identify studies, literature searches were conducted on 13th Oct 2024 across six electronic databases: CINAHL, Cochrane, Embase, MEDLINE, PsycINFO, and Scopus. Publication dates ranged from 1980 to October 2024. Two reviewers (S.M., So.M.) also completed a hand search of reference lists in eligible full-text articles.

### 2.2. Search Strategy

The creation of a structured and comprehensive search strategy was guided by the PICO framework [33]. Electronic search strategies were completed in all six electronic databases using free text and specific subheadings (i.e., MeSH and Thesaurus terms). Four strings of terms were combined for ‘cerebral palsy’, ‘swallow* disorder’ OR ‘feed* difficult*’ OR ‘feed* disorder’, ‘child’, ‘behavioural intervention’, and associated subject headings. All of the retrieved articles were imported into the reference management software Endnote 20 and then exported into Covidence for screening and management.

### 2.3. Inclusion and Exclusion Criteria

Studies were eligible if they (i) included participants aged 0–18 yrs, (ii) required a diagnosis of CP and OPD, (iii) involved delivery of a behavioural intervention that focussed on feeding, eating, drinking, and/or swallowing skills/impairment (OPD), (iv) provided a description of the intervention related to contents and dosage, and (v) clearly referred to using a randomised controlled trial (RCT) design in the title or abstract. Exclusion criteria included (i) lack of clarity as to whether the participant had CP and/or OPD, (ii) provision of an intervention name but not a description of the treatment components (content and dosage), and (iii) papers published before 1980 (due to advances in research methodological practices).

### 2.4. Data Collection Process

A data extraction tool was developed using Excel, then trialled and further modified to create a final robust data extraction form. Three reviewers (B.M.A., M.M., S.M.) independently used this template to extract and tabulate the data on the following variables: purpose/aim of study, age and sex of participants, total number of participants, motoric description of CP, intervention description, outcome measures, and treatment outcomes. Turkstra’s framework [34] was used to describe the treatment components of the intervention. The *target behaviour*/*target(s)* was defined as the specific aspect of functioning selected and intended to change as a result of the treatment; *active ingredients* are specific clinician-directed actions taken to affect a change in the target behaviour; and the *mechanism of action* is the hypothesised means in which the treatment is intended to exert its effects [34,35]. Primary outcomes of interest related to feeding, eating, drinking, and/or swallowing. During data collection, data points across all studies were extracted.

### 2.5. Data, Items, and Synthesis of Results

Two reviewers (S.M., So.M.) independently applied the selection criteria initially to titles and abstracts, and then original articles, to assess for eligibility. To ensure rating accuracy, three group sessions were held and attended by four team members (C.-A.M., M.M., S.M., So.M.) to discuss the ratings of fifty randomly selected records to achieve consensus before rating the remaining abstracts. Any differences in opinion regarding inclusion were mostly resolved through consensus by two team members (S.M., So.M.). A third member of the research team (M.M. or C.-A.M.) was consulted if disagreement occurred, until a final decision regarding inclusion was reached. To ensure a comprehensive search strategy, the criterion of an RCT was not applied until the stage of full-text screening. The risk-of-bias assessment was performed at a study level; with the Revised Cochrane Risk of Bias tool (ROB-2) [36] used by two independent researchers (M.M., B.McA.) to assess the methodological quality of the included studies. There was 100% agreement across each of the five domains, and consensus was therefore reached without the need to involve a third party. The main summary measures for assessing treatment outcomes were effect sizes and significance of findings. A meta-analysis could not be performed, due to ineligibility of included studies, and a narrative method was therefore used to synthesise the data for the key study variables, study quality, and risk of bias. Tabular and graphical formats were used in the reporting of the results.

## 3. Results

### 3.1. Study Selection

**Figure 1:**
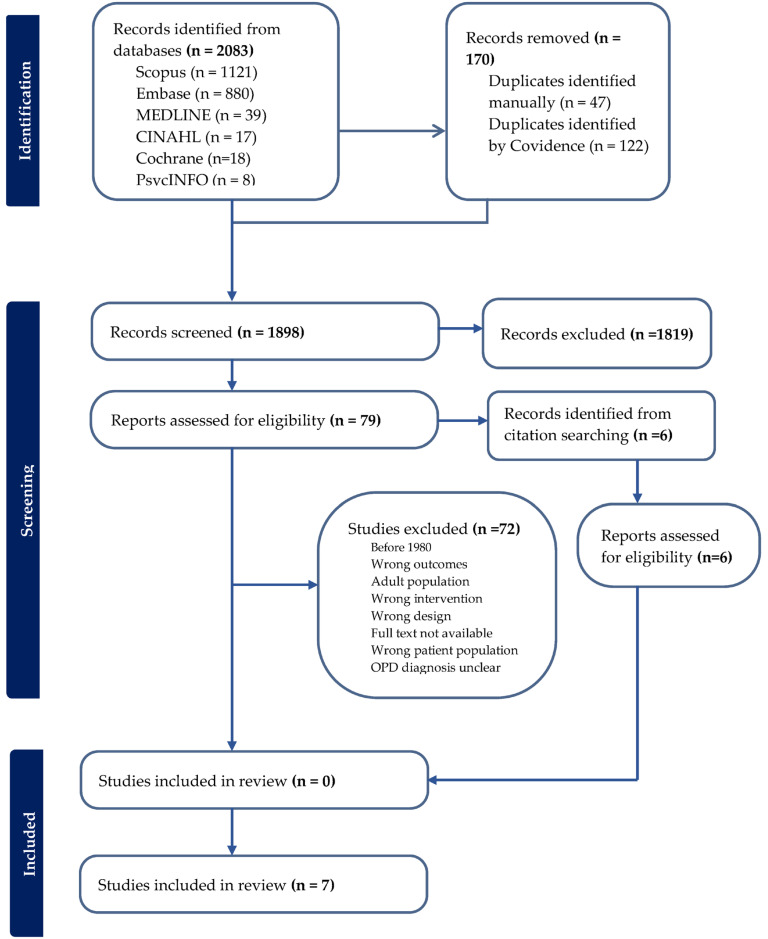
Flow diagram of the reviewing process according to PRISMA.

An initial yield of 2083 studies was retrieved across six databases (CINAHL (*n* = 17)*,* Cochrane (*n* = 18), Embase (*n* = 880), MEDLINE (*n* = 39), PsychINFO (*n* = 8)*,* and Scopus (*n* = 1121)). After removal of duplicate titles and abstracts, a total of 1913 records remained. Following title and abstract screening, 79 original articles were identified, and the full-text records were examined to verify that they met all inclusion criteria. A further six studies were identified from hand searching of these full-text records and were sourced to assess eligibility. Finally, seven RCTs published since 2017 were included. Figure 1 presents the PRISMA flow diagram.

### 3.2. Description of Studies

All seven included studies are described in detail in Table 1 and Table 2 [37,38,39,40,41,42,43]. Three studies had a total sample greater than 50 participants, while four studies had fewer than 40 participants in their sample. In Table 1, information on the study characteristics is presented and includes a definition of OPD, tools/methods used to diagnose OPD, reporting of OPD severity, reported description of participants in the sample, and the intervention group types. A description of participants’ ages, sex, and CP is provided for all study groups in Table 1. Detailed information is provided in Table 2 on the treatment components: the intervention, target behaviour, mechanism of action, primary outcomes of interest, outcome measures, and all treatment outcomes reported in each included study.

#### 3.2.1. Participants (See Table 1)

The seven included studies involved a total of 329 participants with CP (Males, *n* = 182; Females, *n* = 147) who received a type of behavioural intervention to treat their OPD (see Table 1). The participants’ age group ranged from age 9.5 months (SD = 2.03) to 10.6 yrs, with no studies focussed on adolescence. A description of CP was not reported in two studies and there was variability in methods of description across studies (GMFCS level, topography, nature of pathological impairment, etc.). Spastic quadriplegia was the most frequently reported CP sub-classification (*n* = 64), followed by hemiplegia (*n* = 20).

#### 3.2.2. Outcomes and Outcome Measures (See Table 1 and Table 2)

There were a range of outcomes and outcome measures [44,45,46,47,48,49,50,51,52,53,54,55,56,57,58] reported across the seven studies. Sixteen outcomes targeted a body functions and structures (BFS) level of functioning in six studies, and six activity-level outcomes were targeted in five studies. None of the studies assessed outcomes related to participation. Three studies included a quality of life-related outcome. Outcomes targeting a BFS level focussed largely on either the oral preparatory or oral phases of swallowing, e.g., lip and tongue movements. Other primary-related BFS-level outcomes included drooling, weight, physical growth, and negative aspects of functioning related to swallowing, e.g., vomiting. Activity-level outcomes included those primarily focussed on the tasks of feeding, eating, chewing, drinking, and/or swallowing. A range of outcome measures were employed to measure treatment effects in the studies (see Table 1). The most frequently used outcome measures were the Schedule of Oral Motor Assessment (*n* = 3) and the Oral Motor Assessment Scale (*n* = 2) [49,53].

#### 3.2.3. Behavioural Intervention Groups (Table 1)

Each study included a comparison group that received an alternative treatment targeting their OPD. Terms used to describe the comparison included traditional or conventional therapy, sham treatment, or standard care.

#### 3.2.4. Interventions and Treatment Components (Table 2)

Most studies used a combination of behavioural interventions to treat OPD (*n* = 6). Four of the seven studies used a combination of compensatory, direct, and indirect skills training. Five studies used direct skill training techniques. Oral sensorimotor therapy (OSMT)), used interchangeably with the terms oral motor therapy and oral sensorimotor stimulation, was the most frequently trialled intervention (*n* = 4), followed by a form of neurodevelopmental treatment (NDT) (*n* = 2). Three studies reported embedding traditional behavioural techniques of reinforcement through verbal and visual means. The active ingredients including the content and dosage information were largely well described, and the setting information was provided for each study. The latter included university clinic, hospital, outpatient department, and home/telehealth/online settings. The target behaviour was not clearly outlined in three studies, and the mechanism of action was not mentioned in two studies. No study reported treatment fidelity.

### 3.3. Methodological Quality

**Figure 2:**
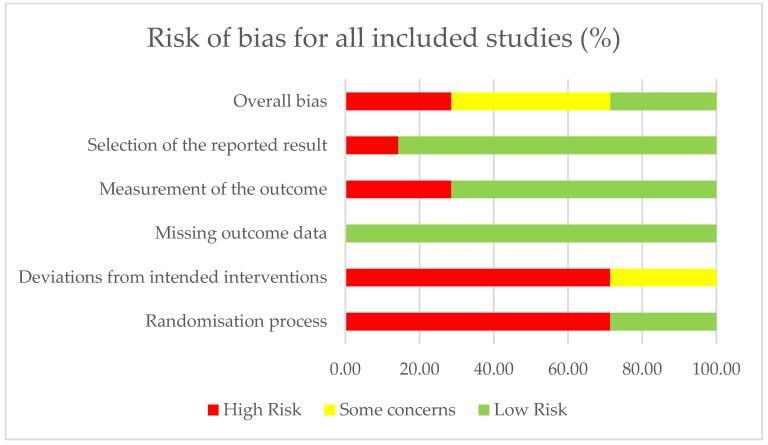
Risk of bias summary for all included studies (*n* = 7) in accordance with ROB-2.

The methodological quality of the included RCTs was assessed using the ROB-2 tool [36]. Table 3 and Figure 2 present the risk-of-bias summary per domain for individual studies and for all included studies. Only two studies showed a low risk of bias overall, with one domain having some concerns; five studies were deemed at high risk of bias as they each had a rating of at least two domains for ‘high bias’. In five studies, there was either unclear or absent reporting regarding allocation concealment or a lack of reporting on the random sequence generation method, indicating a higher selection bias. Blinding of participants and personnel was also absent or not clearly reported in five studies. Blinding of outcome assessment was not reported in two studies, potentially leading to increased detection bias. While two pilot RCTs had an overall low risk of bias, both studies had fewer than 10 participants in each of the respective experimental and control groups, indicating that a meta-analysis would not yield reliable results [59,60].

## 4. Discussion

This study aimed to determine the effectiveness of behavioural interventions in the treatment of OPD in children with CP.

### 4.1. Lack of Robust RCTs

Considering the serious impact of OPD on the health and quality of life of individual children with CP, the small number of high-quality RCTs being undertaken is a cause for concern. RCTs and systematic reviews of such trials provide the most reliable evidence about the effects of healthcare treatments [61]. In total, seven behavioural RCTs in the paediatric population of CP, which involved a total of 329 participants, were identified. Our review highlighted that there was considerable clinical heterogeneity amongst the seven included RCTs, which limits the ability to generalise the findings to a real-world context [62]. Clinical heterogeneity can be viewed as differences in participant characteristics, outcome measures, and intervention characteristics, including dose [63]. We found omitted or variable participant baseline data, in relation to CP sub-classification and/or OPD severity, which may have affected the participants’ intervention responses, and therefore, the study outcomes [64]. In addition, the variability in outcome measures used across studies, e.g., related to trunk control vs. oral phase of swallowing, makes it difficult to directly compare treatment results [65]. Whilst we found that mostly treatment combinations of compensatory and skills training occurred across studies, the individual interventions within these combinations, and by extension, their active ingredients, varied, e.g., chewing therapy vs. action observation therapy. Variations in active ingredients infer a different set of treatment components were used [34], and the treatments therefore are not directly comparable. Dosage was also set at different intensities across intervention studies, e.g., number of exercise trials vs. number of weeks. Poor fidelity reporting further complicates our understanding of dosage, as potential variations in intervention delivery can affect the participants’ exposure to the treatment components or dose [64,66]. The collective variability across participants, treatment components, and outcome measures significantly influences the reliability of the treatment effect [62,67] and does not allow for the generalisability of findings [68].

The clinical heterogeneity found in this review is further compounded by the high risk of bias found in five studies. A hallmark characteristic of an RCT design is random allocation and blinding [60]. However, only two studies included sufficient reporting on the processes of randomisation and blinding. Two studies also had a high risk of bias for outcome measurement, thus undermining the ability to draw causal inferences regarding the intervention’s true effects [60]. The two pilot RCTs that demonstrated overall low-level bias are a promising indication that high standards can be achieved; however, the inclusion of larger samples and increased consistency in reporting are needed to facilitate the conduct of a meta-analysis.

### 4.2. Progress in the Research Field of OPD in CP

Of note, the seven included studies in this review were published in the past eight years, providing contemporary studies from which to draw evidence; however, this still strongly suggests that treatment for OPD in paediatric CP is an under-researched area. Whilst outcomes in the seven studies focused on impaired BFS-level outcomes, a positive was that five studies outlined how activity-level outcomes were targeted, focusing on more global aspects of functioning related to swallowing. Previous research has highlighted a predominant focus on compensatory methods when implementing behavioural methods to treat OPD in children with CP, largely addressing impairment-level outcomes [21]. It is encouraging that direct skill training was the primary focus in five studies, and in four of the seven studies, a combination of skill training and compensatory behavioural techniques was used. The latter is important as it aligns with best-practice principles when developing effective behavioural interventions [69]. OPD is a complex behaviour; therefore, a single intervention approach used in isolation is unlikely to produce a significant change in behaviour. In our review, we found that the active ingredients of the interventions were largely well-reported. Reporting this detailed information on content and dosage allows a greater opportunity to test how treatment components are linked to the mechanism of action(s), which is a prelude to evaluating clinical significance [70]. Importantly, five studies used a patient-reported outcome measure or quality-of-life measure, and three studies utilised a test to determine effect sizes in order to help ascertain clinical significance [71].

### 4.3. What Are the Important Elements of a Behavioural Intervention in the Treatment of OPD in CP?

This review highlighted fundamental issues about implementing behavioural interventions to treat OPD in children with CP. First, a functional analysis of the baseline condition, that is, feeding, eating, drinking and/or swallowing skills, is needed to enhance our understanding of the primary aetiological factors of the child’s condition [72,73], in this case, OPD. In a novel contribution, our review highlighted a range of relevant tools used to assess OPD or OPD-related behaviour. However, the links between the identified individual impairments from those measures and their likely effects on swallowing function were not adequately described. Clearly identifying and reporting the antecedent behaviours and their consequences helps to hypothesise regarding potential causation, supporting the development of a more tailored evidence-based intervention [22,74]. For example, a child with CP and a GMFCS level V has poor head control secondary to when poorly positioning in his customised seating, leading to an increased frequency of mouth opening and loss of food/fluid. The antecedents are sub-optimal positioning and poor head control, leading to consequences of increased tongue thrusting, reduced lip closure, and increased delay in triggering pharyngeal swallow (which increases the risk of premature spillage into the airway and subsequent aspiration). We hypothesise that poor seating is a controlling variable and maintaining factor in decreasing performance at the oral preparatory and oral stages of swallowing, thus increasing the risk of aspiration. An occupational therapy referral for seating review is prompted; the child receives a new customised seating system and as a result has a more optimal position for safer and more effective swallowing.

As part of a functional analysis, sufficient information must be provided for the baseline characteristics of the individual participant data, including the diagnosis and severity of the condition to be identified. In our review, two of the seven studies [40,43] did not provide a CP description beyond diagnosis. Further, only two studies [38,42] used the EDACS tool [58] to describe levels of ability regarding safety and efficiency in eating, drinking and swallowing and no study reported baseline OPD severity. Reporting this additional information on CP and OPD diagnoses, and their severities, will facilitate increased accuracy in identifying antecedent behaviours, in turn, optimising chances of success in developing an effective functional behavioural treatment [22,72,73,74].

Clear reporting of the target behaviour of the intervention is needed in order to know which specific aspect of functioning is intended to change because of the treatment [34,74]. Our study highlighted that the target behaviour(s) of the intervention was not always clearly articulated in each study, separately from outcomes. Detailing an operational definition of the target behaviour outlines what is needed from the recipient of the intervention to signify that event [22,75], facilitating increased transparency and accuracy in the recording of that behaviour. Additionally, the mechanisms of action were not consistently reported across studies. Outlining the mechanisms of action(s) provides information on the intrapersonal processes that must change in order to achieve a clinical response [76]. Providing details regarding the target behaviours and mechanisms of actions can contribute to formulating a hypothesis as to how the treatment might be effective. Without this theory-driven approach, we cannot identify the key components that render the treatment effective [34,77].

The consistent reporting of treatment fidelity is of paramount importance as higher levels of treatment fidelity are associated with higher gains attributable to intervention [78]. As a positive, the largely well-reported active ingredients of behavioural interventions in the included studies ‘partly’ ensure treatment fidelity [75], but levels of adherence still need to be reported [79]. Finally, OPD is complex, with multiple determinants, thus warranting equally complex interventions with coordinated input across many different disciplines. Detailed information on intervention agent, e.g., discipline, and/or setting information was inconsistently reported across the RCTs. The provision of more detailed information on the involvement of the team, in addition to specifying the intervention agent and setting, will facilitate an enhanced understanding of how treatments might be effective if significant results are found.

### 4.4. Strengths and Limitations

This systematic review aimed to answer a specific focused clinical intervention question. Finding only seven RCTs despite a comprehensive systematic search in six databases is an indication that this is an under-researched area. While papers in all languages were included to minimise publication bias, four studies from Chinese journals could not be retrieved and these studies may have contained relevant experimental data. Data on race and ethnicity were not collected, which may reduce the generalisability of the findings. As we limited our focus to RCTs only, since these represent the highest level of evidence, other potentially relevant experimental research may have been excluded.

Future methodologically robust and larger RCTs that minimise clinical heterogeneity and provide detail on the functional analysis of OPD (to outline primary determinants) are urgently needed. Clearly describing the treatment components may also help to identify the critical components that underlie its efficacy [34,69], leading to advanced insights into how received treatment improves health [80]. The reporting of treatment fidelity is also a priority in future OPD-focused studies, to facilitate higher validity and translation into clinical practice [81]. Statistical testing alone is not sufficient to evaluate a clinically relevant effect. Standardised effect sizes are recommended when studies use different measurement scales to facilitate comparison between studies and foster completion of meta-analysis [82]. Collaboration between experts on how to support the implementation of such high-quality RCTs would help to accelerate progress. By taking these measures, transparency in research reporting will be facilitated and help to advance progress in the field.

## 5. Conclusions

Progress in proving the effectiveness of behavioural interventions to treat OPD in children with CP is slow. Despite the serious health repercussions for children with CP and OPD, it remains the case that low-level evidence supports the use of behavioural interventions to treat OPD in children with CP. Rigorously designed RCTs with larger samples of children with CP and OPD are urgently needed, to evaluate the effectiveness of behavioural interventions across the developmental childhood phase. In future research, consistency in describing a functional analysis of swallowing in OPD together with reporting theoretical treatment component-related data and treatment fidelity is a priority.

## Figures and Tables

**Table 1:** Baseline characteristics of studies on behavioural interventions for children with CP with oropharyngeal dysphagia.

Study Country	OPD Definition Method of OPD Assessment OPD Severity (at Baseline)	Sample (N) Groups (*n*) ^a^	Group Descriptive (Mean ± SD) (Age, Sex, CP Description)
Abd-Elmonem et al. 2021 [37] Egypt	***OPD definition*:** ‘Having at least a problem of oral motor functions (sucking, drooling, swallowing) ***Tool for OPD*/*OPD-related behaviour:*** OMAS ***OPD severity*:** Not defined	***n* = 64** Intervention group = OSMS + 10 mins of rest + 90 min of NDT (*n* = 32) Control group = 90 min of NDT (*n* = 32)	Intervention group/control groups ***Age:*** 29.65 +/− 8.09 mths/29.18 +/− 7.97 mths ***M*/*F:*** M, *n* = 30, F, *n* = 34 ***CP description:*** Spastic quadriplegia (*n* = 64) **GMFCS Level**, median (IQR): EG = 4 (5.4) CG = 4 (5.4)
Acar et al. 2022 [38] Turkey	***OPD definition*:** ‘Difficulty in feeding/swallowing’ ***Tool for OPD*/*OPD-related behaviour:*** *SOMA* ***OPD severity*:** Not defined; outlined ‘dysfunctions’ re. specific behaviours in SOMA	***n* = 40** Intervention group = OMIS + NRCT + NDT-B (neck + trunk stabilisation ex.) Visual, verbal, + proprioceptive feedback was given to the child (*n* = 20) Control group = OMIS + NRCT (*n* = 20)	Intervention group/control group ***Age*:** 3.30 +/− 0.76/2.97 +/− 0.91 ***M*/*F:*** M, *n* = 8, F, *n* = 12/M, *n* = 11, F, *n* = 9 ***CP description:*** Diplegia: 3 vs. 1; Hemiplegia: 2 vs. 2; Quadriplegia: 8 vs. 10; Dyskinesia: 1 vs. 2; Hypotonic: 6 vs. 5 **GMFCS Level** Level I: 4 vs. 2; Level III: 2 vs. 0; Level IV: 4 vs. 4; Level V: 10 vs. 14 Mini-Macs Level Level II: 4 vs. 3; Level III: 5 vs. 5; Level IV: 3 vs. 2; Level V: 7 vs. 9 **EDACS Level** (*n* = 16) (3.25 ± 1.12)/(*n* = 11) (3.27 ± 0.78) Level I: 1 vs. 0; Level II: 3 vs. 1; Level III: 5 vs. 7; Level IV: 5 vs. 2; Level V: 2 vs. 1
Akalthun et al. 2023 [39] Turkey	***OPD definition*:** ‘Who had oropharyngeal dysphagia symptoms or findings and was subsequently hospitalised and rehabilitated’ ***Tool for OPD*/*OPD-related behaviour****:* Observation/recording of mealtime; Mealtime length; FOIS; Pulse oximetry ***OPD severity*:** Not defined	***n* = 101** Intervention group = Kinesiotaping applied with stretching to suprahyoid region (*n* = 54) Sham group = Kinesio tape was applied without stretching to the suprahyoid region (*n* = 47) All pcpts had education on 4 items of care related to swallowing characteristics of the children	Intervention group/control group ***Age*:** 50.4 +/− 17.4 mths/47.9 +/− 18.6 mths ***M*/*F****:* M, *n* = 34, F, *n* = 20/M, *n* = 25, F, *n* = 22 ***CP description:*** **GMFCS Level:** 4.1 +/− 1.1 vs. 3.9 +/− 1.1 Hemiplegia: *n* = 7/*n* = 9 Diplegia: *n* = 13/*n* = 11 Triplegia: *n* = 24/*n* = 21 Tetraplegia: *n* = 10/*n* = 6
Khamis et al. 2023 [40] Australia	***OPD definition*:** OPD as determined by a CFE ***Tool for OPD*/*OPD-related behaviour****: CFE*, *SOMA, IDSSI, FOISi* ***OPD severity*:** Not defined; minimum of 20% of nutrition consumed orally; outlined dysfunctional behaviours on SOMA, IDSSI levels, and FOISi levels outlined	***n* = 14** Intervention group = babiEAT: twice-weekly for 4 weeks followed by once-weekly home visits for 8 wks (total = 12 wks) (*n* = 8) Standard care group = As per service protocol, which varies, typically weekly to monthly (12 wks) (*n* = 6)	Intervention group/standard care group ***Age*:** 9.63 (SD = 2.33 mths)/9.33 (SD = 1.75) ***M*/*F****:* M, *n* = 4, F, *n* = 4/M, *n* = 2, F, *n* = 4 ***CP description:*** Not described beyond CP diagnosis (babies originally identified as at high risk of CP)
Manzoor et al. 2024 [41] Pakistan	***OPD definition*:** ‘Feeding and swallowing disorders’ ***Tool for OPD*/*OPD-related behaviour:*** *FOMS and DSFS (for drooling)* ***OPD severity*:** Not defined	***n* = 10** Intervention group = OMT (*n* = 5) Conventional SP group = (*n* = 5)	Intervention group/standard care group ***Age*:** 5.66 +/− 2.02 yrs/5.78 +/− 1.91 yrs ***M*/*F:*** M, *n* = 4, F, *n* = 1/M, *n* = 2, F, *n* = 3 ***CP description:*** Spastic, *n* = 4 vs. *n* = 3; Ataxic; *n* = 0 vs. *n* = 1; Athetoid, *n* = 1 vs/*n* = 1
Mokhlesin et al. 2024 [42] Iran	***OPD definition*:** ‘Impairment of oral phase of swallowing’; used DDS ***Tool for OPD*/*OPD-related behaviour:*** OMAS, SOMA, FS-IS, Pedi-EAT ***OPD severity*:** Not defined	***n* = 20** Intervention group = AOT, OSMT, and reinforcement (*n* = 10) Control group = Sham tx, OSMT, and reinforcement (*n* = 10)	Intervention group/control group ***Age*:** 8.6 +/− 2 yrs/7.6 +/− 1.2 yrs ***M*/*F****:* M, *n* = 7, F, *n* = 3/M, *n* = 8, F, *n* = 2 ***CP description:*** **GMFCS Level:** Level III: 5/6; Level IV: 5/4 **EDACS Level:** Level II: 4/3; Level III: 6/7
Serel Arslan et al. 2017 [43] Turkey	***OPD definition*:** ‘Children with CP who had complaints about chewing function and could not manage solid food intake over the age of 18 mths’ ***Tool for OPD*/*OPD-related behaviour:*** OMA; BPFAS, KCPS (chewing) ***OPD severity*:** Not defined. Parent reports of solid food refusal, holding food in mouth, trying to mash the food between tongue and palate, choking, gagging, and pushing food out of mouth	***n* = 80** Intervention group = FuCT (*n* = 50) Control group = Traditional OME (passive and active lip + tongue exercises (*n* = 30)	Intervention group/control group ***Age*:** 3.5 +/− 1.9 yrs/3.4 +/− 2.3 yrs ***M*/*F:*** M, *n* = 31, F, *n* = 19/M, *n* = 16, F, *n* = 14 ***CP description:*** Not described beyond CP diagnosis

^a^ Terminology used by author(s). *Notes:* AOT = action observation therapy; CG = control group; CP = cerebral palsy; BPFAS = Behavioral Pediatrics Feeding Assessment Scale; CFE = clinical feeding evaluation; DDS = Dysphagia Disorders Survey, DSFS = Drooling Severity & Frequency Scale; F = female; FOIS = Functional Oral Intake Scale; FOISi = Functional Oral Intake Scale for Infants; FOMS = Feeding Oral Motor Scale; FS-IS = The Feeding Swallowing Impact Scale; FuCT = functional chewing therapy; IDSSI = International Dysphagia Diet Standard Initiative; IQR = interquartile range; KCPS = Karaduman Chewing Performance Scale; OMA = oral motor assessment; OMAS = Oral Motor Assessment Scale; OMEs = oral motor exercises; OMIS = oral motor intervention strategies; OMT = oral motor therapy; OPD = oropharyngeal dysphagia; OSMS = oral sensorimotor stimulation; OSMT = oral sensorimotor therapy; M = male; mins = minutes; mths = months; NRCT = nutrition-related caregiver training; NDT = neurodevelopmental treatment; NDT- B = neurodevelopmental therapy—Bobath; pcpts = participants; SOMA = Schedule of Oral Motor Assessment; tx = treatment; wks = weeks; yrs= years. See Section Abbreviations.

**Table 2:** Outcome of behavioural interventions for children with CP and oropharyngeal dysphagia.

Author and Purpose of Study	Intervention and Target Behaviour Outcomes ICF-CY Classification	Active Ingredients *Agent of Intervention* *Setting* *Behavioural Intervention Sub-Type* *Content (Exercises and Content)* *Dosage (Trials, Regularity, Periodicity, Timeframe)*	Mechanism of Action *Hypothesised Theory*	Mechanism of Action *Sub-Type*	Outcome Measures	Treatment Outcomes *Primary Outcomes*
Abd-Elmonem et al. 2021 [37] *To explore the effect of Oral Sensorimotor Stimulation on oral motor skills* *and weight gain in children with spastic quadriplegia*	Intervention: Oral Sensorimotor Stimulation (based on Fucile protocol). Target Behaviour: To decrease hypersensitivity of oral structures, increase jaw movement, and reinforce muscle strength, improve tongue movement and enhance Oral Motor (OM) organisation Outcomes **Body Functions and Structures Level** OM skills Segmental trunk control Physical growth Gross Motor function	Intervention Group: **Agent:** Certified physical therapists (previously instructed regarding the aims and the protocol manoeuvres) **Setting:** Outpatient clinic **Behavioural Intervention Sub-Type:** skills training--indirect **Content:** Neurodevelopmental Therapy (NDT)-based sequenced trunk co-activation exercises + Oral Sensorimotor Stimulation (OSMS) **Dosage:** 20 min Oral Sensorimotor Stimulation, + 10 min rest + 90 min NDT, 5 days/week for 4 successive months Duration of each OSMS given Control Group **Agent:** Certified physical therapists (previously instructed regarding the aims and the protocol manoeuvres) **Setting:** Outpatient clinic **Content:** NDT-based sequenced trunk co-activation exercises **Dosage:** 90 min 5 days/week for 4 successive months	NDT: To regain typical movement by prohibiting abnormal tone, promoting postural reactions, and enhancing postural mechanisms OSMS: Decrease hypersensitivity of oral structures, increase jaw movement, reinforce muscle strength, improve tongue movement, and enhance oral motor organisation *Overall, relationship to swallowing not explained*	Changing output of organ systems (methods to +truncal control to support better posture during mealtime)	OMAS SATCo Body mass-weight GMFM-88	Significant difference in post-treatment OMAS scores of the experimental group compared with the control group (*p* > 0.001) * and significant increase in OMAS scores of the experimental group compared with the pre-treatment scores (*p* > 0.001) *. Significant increase in static, active, and reactive scores of SATCo in the experimental and control groups (*p* > 0.001) *. However, post-treatment comparison between groups revealed no significant difference in SATCo scores (*p* > 0.05). Significant increase in weight (kg) (*p* > 0.001) * (MD (95% CI) = 2.42, −2.57; −2.26) and GMFM-88 in the experimental group post-treatment (MD (95% CI) = 38.17 +/− 3.6 (*p* = 0.001). No significant change in weight of the control group (*p* > 0.05), while there was a significant increase in GMFM-88 post-treatment mean values compared with the pre-treatment (*p* > 0.001) *. Post-treatment comparisons revealed a significant difference in weight (kg) in favour of the experimental group compared with that of the control group (*p* < 0.001), while there was no significant difference in GMFM-88 between groups’ post-treatment mean values (*p* > 0.05). * Significant difference reported in text; however, symbol error when reporting significance.
Acar et al. 2022 [38] *To investigate the effects of the structured Neurodevelopmental Therapy Method—Bobath* (*NDT-B) on the feeding and swallowing activity of patients with CP and feeding difficulties*	Intervention: NDT-B, Oral Motor Intervention Strategies (OSMS), Nutrition Related Caregiver Training (NRCT) Target Behaviour: not clear To maintain pelvic stability to support trunk control and finally influence head control, jaw stability and tongue/lip movement Outcomes: **QOL** **Body Function and Structure Level-** not clear Truncal control/power **Activity-Level** Feeding and swallowing activity	Intervention group: OMIS + NRCT + NDT-B Group **Agent:** PT **Setting:** University hospital **Behavioural Intervention Sub-Type:** Compensatory, skills training—indirect, skills training—direct **Content:** NDT-B-based neck and trunk stabilisation exercises, OMIS, NRCT feedback and modifications given, checklists for families to check they were performing exercises **Dosage**: NDT-B = 45 min session, 2 days/week for 6 weeks, NRCT home program for 6 weeks, standardised treatment protocol Control group: OMIS + NRCT Group: **Agent:** PT **Setting:** University hospital **Content:** OMIS + NCRT **Dosage:** NDT-B = 45 min session, 2 days/week for 6 weeks, NRCT home program for 6 weeks	Child’s head position influences swallowing during feeding and reduces risk of aspiration. NDT-B and OMIS hypothesised to have an effect on the feeding and swallowing activity. Exercises applied expected to increase the control of the trunk, reduce the duration of mealtime, reduce pain and discomfort after feeding, and increase the QOL	NDT-B- changing output of organ systems, increased trunk and postural control NRCT- Changing cognitive/affective representations and improving efficiency of skilled performance OMIS- changing output of organ systems and improving the quality, speed, efficiency, or automisation of skilled performance at function or activity level	TIS SOMA PedsQL	Greater improvement in static sitting balance and total TIS scores in the OMIS + NRCT + NDT-B group compared to the OMIS + NRCT group. Group effect was not significant on any scales. Increase in SOMA post-treatment results on items of trainer cup, bottle, and puree in both groups; however, improvement was greater in the OMIS + NRCT + NDT-B group. OMIS + NRCT + NDT-B group was superior in the trainer cup and puree subcategories (*p* = 0.05). Group effect was not significant on any scale. The QOL increased in both groups by affecting the physical functioning parameters (participating in active play and exercise, reducing aches and pains, etc.), but no superiority was found between the groups.
Akalthun et al. 2023 [39] *To investigate the short- and long-term effects of kinesiotape on dysphagia in children with CP*	Intervention: Application of Y-type kinesiotape Target Behaviour: Not clear Outcomes: **Mealtime** (**length** **Oral Intake** **Body Function and Structure Level** Lip + tongue motions Coughing Choking Retching Vomiting **Activity-level** Eating + drinking	Intervention Group: **Agent:** Tape applied by specialist, profession not specified **Setting:** Hospital **Behavioural Intervention Sub-Type:** Unclear **Content:** Y-type kinesiotape, specifics of placement outlined on pg. 436 **Dosage:** Applied 2 times/week for 6 weeks; 3 days tape, 1 day rest, and 3 days tape Sham Group: **Agent:** Tape applied by specialist, profession not specified **Setting:** Hospital **Content:** Type of tape not specified, applied in different area without stretching **Dosage:** Applied 2 times/week for 6 weeks; 3 days tape, 1 day rest, and 3 days tape	Not clear	Not reported	FOIS Likert scale—family satisfaction Mealtime length in minutes Pulse oximetry Observation and video recording of mealtime	Drooling, weak tongue movement, chewing difficulty, coughing/choking, and retching/vomiting, as well as FOIS score and mealtime length, significantly improved in the kinesiotape group at 6 and 18 weeks compared to pre-treatment scores (*p* < 0.017). Although the 18-week values decreased slightly compared to the 6th week, there was no significant difference (*p* > 0.017). In the sham group, there was no significant difference in any parameter at 6 and 18 weeks compared to pre-treatment (*p* > 0.017). The kinesiotape group’s satisfaction level was significantly greater compared to the sham group (*p* = 0.008). Patients were more likely to answer “much better” or “slightly better” in the kinesiotape group at 6 weeks compared to the sham group (*p* = 0.003).
Khamis et al. 2023 [40] *To assess the feasibility and acceptability of the baby intensive Early Active Treatment (babiEAT) and standard care feeding interventions and explore the preliminary efficacy of babiEAT versus standard care on OPD, health, and caregiver feeding-related QoL in infants at high risk of CP with OPD*	Intervention: babiEAT Target Behaviour: Not clear Outcomes: **Feasibility and accept-** **ability of both feeding therapy programs** **Feeding Related Quality of Life** **Body Functions and Structures Level** Oral feeding efficiency Weight Number. of chest infections + hospitalisations (in 3 months before study) **Activity-Level** Feeding and swallowing	Intervention group: babiEAT **Agent:** SLT **Setting:** Home/online **Behavioural Intervention Sub-Type:** Compensatory, skills training—indirect, skills training—direct **Content:** Caregivers practice these skills at snack time for 15 min, 3 times/daily, incorporating praise, comments on performance, singing, explorative play with hands, and preferred flavours. Caregivers asked to keep logbook **Dosage:** 60 min session 2 times/week for 4 weeks followed by 60 min session 1 time/week for 8 weeks. Total 12-week duration Standard Care: **Agent:** SLT **Setting:** Home/clinic/online **Content:** Variable dependent on clinician and service protocol. Caregivers asked to keep logbook **Dosage:** Average number of treatment hours and home program were significantly less than Ix group	Direct intervention following neuroplasticity and motor learning principles (which propose that early, intense practice that is challenging and as close to the task as possible produces the best outcomes) and incorporation of food or fluid stimuli	Changing output of organ systems and improving quality of skilled performance at either a function or activity level	FIPQ FRQoL FS-IS GAS SOMA IDDSI Level FOISi % of total volume consumed during first 5 min of mealtime Mealtime duration Number of compensatory strategies used Weight for age Number of chest infections and hospitalisation in 3 months pre- and during intervention	babiEAT intervention perceived by caregivers to be more “effective” than standard care (*p* = 0.048) and babiEAT caregivers more likely “to recommend this feeding therapy program to a friend”. Statistically significant difference in feeding efficiency with fluids in favour of the babiEAT group (*p* = 0.03). No significant difference in SOMA scores for fluids (bottle *p* = 0.31; trainer cup (straw) *p* = 0.19; cup *p* = 0.09); however, a statistically significant within-group reduction in compensations for cup drinking was noted for babiEAT participants compared to the standard care group (*p* = 0.02). No statically significant difference in the achievement of GAS goals for fluids (*p* = 0.053); mean for participants in the babiEAT group increased 2 SD from baseline and surpassed a T-score of 50, indicating goal achievement—a clinically significant outcome. Mean T-score for participants in the standard care group did not reach 50, indicating goals were not achieved. No significant differences in the % of solids consumed in the first 5 min (feeding efficiency) (*p* = 0.63) or GAS goals for solids (*p* = 0.30) between babiEAT and standard care participants. Mean solids T-score for babiEAT group improved over 2 SD from baseline and surpassed a T-score of 50 representing goal attainment, while the mean for those who received SC remained under 50, indicating goals were not reached; a clinically significant between-group difference. babiEAT participants made significantly more progress in both the SOMA Solids subtest (*p* = 0.047) and the recommended IDDSI level for solids (*p* = 0.02). FOISi ratings demonstrate a significant between-group difference in oral intake (*p* = 0.02). Mean duration of mealtimes for the babiEAT group reduced to less than 30 min, despite the babiEAT mean being higher prior to the intervention. No statistically significant between-group difference was found in Z-score weight measures (*p* = 0.51). babiEAT parents reported significantly higher QoL in all three FS-IS subtests than standard care parents.
Manzoor et al. 2024 [41] *To assess the effects of oral motor therapy (OMT) in children with CP who have feeding and swallowing difficulties*	Intervention: OMT; exercises designed to improve the strength, coordination, and function of the muscles involved in speech and swallowing. Target Behaviour: to enhance swallowing and chewing functions, with tactile and proprioceptive stimuli used to improve Oral Motor skills. Outcomes: **Body Functions and Structures Level** OM skills Drooling **Activity-Level** Feeding + swallowing skills	Intervention Group: **Agent:** Not reported **Setting:** University campus clinic **Behavioural Intervention Sub-Type:** Skills training—indirect, skills training—direct **Content:** Exercises focused on ‘enhancing swallowing and chewing functions, with tactile and proprioceptive stimuli used to improve oral motor skills’ **Dosage:** Intervention delivered over a 16-week period Control Group: **Agent:** Not reported **Setting:** University campus clinic **Content:** Traditional speech and language therapy; pre-language skill development, manual sign language, gestures, use of picture communication boards, and voice output communication devices, articulation exercises, cognitive therapy, receptive language development, vocabulary building **Dosage:** Intervention delivered over a 16-week period	Exercises designed to improve the strength, coordination, and function of the muscles involved in speech and swallowing	Not reported	FOMS DSFS	Treatment Group: mandible mobility (lateral) improved significantly, with 40% achieving regular mobility post-treatment (*p* = 0.038). Tongue retraction activity showed 100% improvement in the treatment group compared to 20% in the control group post-treatment (*p* = 0.010). Lip protrusion improved to 20% optimal in the treatment group, while the control group showed 60% improvement (*p* = 0.026). Drooling frequency decreased significantly in the treatment group, with 20% showing occasional drooling post-intervention compared to 80% in the control group (*p* = 0.038).
Mokhlesin et al. 2024 [42] *To investigate the impact of Action Observation Training (AOT) on the oral phase of swallowing in children with spastic CP*	Intervention: AOT Target Behaviour: Improve oral phase of swallowing; maintain lip seal around spoon, perform lateral tongue movements, chewing, biting after swallowing and chewing sequence Outcomes: **Feeding-related QOL** **Body Functions and Structures Level** Oral phase of swallowing	Intervention Group: **Agent:** SLP **Setting:** Not reported **Behavioural Intervention sub-type:** Compensatory, skills training—indirect, skills training—direct **Content:** Protocol including action observation, oral sensorimotor therapy, positioning, and reinforcement **Dosage:** 20 min sessions, 1 per day, 5 days/week for 10 weeks Control Group: **Agent:** SLP **Setting:** Not reported **Content:** Protocol including sham treatment, oral sensorimotor therapy, positioning, and reinforcement **Dosage:** 20 min sessions, 1 per day, 5 days/week for 10 weeks	AOT would accelerate motor learning and improve the oral phase of swallowing. Protocol included some motor learning principles. *No specific relation to paediatric CP populations*	Changing output of organ systems (methods to increase truncal control to support better posture during mealtime)	SOMA OMAS Pedi-Eat FS-IS	Significant difference between the two groups in the oral phase of swallowing after the intervention (*p* = 0.03, *Cohen’s d* = 1.07). No significant difference found in the parent-reported scores of the FS-IS and symptoms of feeding problems between the two groups (*p* = 0.07). Significant difference in SOMA scores between both groups at post-treatment assessment (*p* = 0.03) but not significant in follow-up (T3) evaluation (*p* = 0.09)
Serel Arslan et al. 2017 [43] *To investigate the effect of Functional Chewing Training (FuCT) on chewing function in children with CP*	Intervention: FuCT Target Behaviour: Improve chewing function by providing postural alignment, sensory and motor training, and food and environmental adjustments. Outcomes: ***Activity-Level*** Chewing performance (Oral preparatory phase) Feeding performance (from perspective of parents/caregivers)	Intervention group: FuCT **Agent:** Physical therapist **Setting:** University clinic **Behavioural Intervention Sub-Type:** Compensatory, skills training—indirect, skills training—direct **Content:** Impairment-based (positioning, sensory stimulation, chewing exercise), adaptive (food consistency) components. Steps outlined **Dosage:** 5 sets of exercises per day, 5 days/week for 12 weeks as a home programme Control group: Traditional Oral Motor Intervention **Agent:** Physical therapist **Setting:** University clinic **Content:** ‘Traditional oral motor exercises including passive and active exercises of lips and tongue’ **Dosage:** 5 sets exercises per day, 5 days/week for 12 weeks as a home programme	The protocol aimed to ensure functional improvement in chewing function by stimulating and teaching the function	Changing output of organ systems (methods to increase truncal control to support better posture during mealtime)	BPFAS KCPS	Significant improvement observed in KCPS scores at 12 weeks after training in the FuCT group (*p* < 0.001), but no change found in the control group (*p* = 0.07). Significant improvement detected in all parameters of BPFAS at 12 weeks after training in the FuCT group (*p* < 0 001) and in four parameters of BPFAS in the control group (*p* = 0. 02).

Terminology used by author(s). *Notes:* AOT: Action Observation Training; Ax: assessment; babiEAT: baby intensive Early active Treatment; BPFAS: Behavioral Pediatrics Feeding Assessment Scale; CI = confidence intervals; CP: Cerebral Palsy; DSFS: Drooling Severity and Frequency Scale; FIPQ: Feeding Intervention Preferences Questionnaire; FOIS: Functional Oral Intake Scale; FOISi: Functional Oral Intake scale for Infants; FOMS: Feeding Oral Motor Scale; FRQoL: Feeding-Related Quality of Life; FS-IS: Feeding and Swallowing Impact Survey; FSIS: Feeding Swallowing Impact Scale; FuCT: Functional Chewing Training; GAS: Goal Attainment Scale; GM: Gross Motor; GMFM-88: gross motor function measure-88; IDDSI: International Dysphagia Diet Standardisation Initiative; KCPS: Karaduman Chewing Performance Scale; KT: kinesiotaping; MD = mean difference; NC: not clear; NRCT: Nutrition Related Caregiver Training; NDT-B: Neurodevelopmental Therapy Method-Bobath; OM: oral motor; OMAS: Oral Motor Assessment Scale; OMIS: oral motor intervention strategies; OMT: Oral motor therapy; OPD: Oropharyngeal Dysphagia; OSMS: Oral Sensorimotor Stimulation; min = minutes; Pedi-Eat: Pediatric Eating Assessment Tool; PedsQL; Pediatric Quality of Life Inventory; PT: Physiotherapist; QOL: Quality of Life; SATCo: Segmental Assessment of Trunk Control; SD: standard deviation; SLP: Speech-Language Pathologist; SLT: Speech and Language Therapist; SOMA: Schedule for Oral Motor Assessment; TIS: Trunk Impairment Scale; Tx: treatment. See See Section Abbreviations.

**Table 3:** ROB-2 summary table.

	RAND	DEV	MIS_OUT	MEAS_OUT	SEL_REP	OVERALL
Abd-Elmonem et al. [37]	**H**	**H**	**L**	**H**	**L**	**High**
Acar et al. [38]	**H**	**H**	**L**	**L**	**L**	**Some concerns**
Akalthun et al. [39]	**H**	**H**	**L**	**L**	**L**	**Some concerns**
Khamis et al. [40]	**L**	**Some concerns**	**L**	**L**	**L**	**Low**
Manzoor et al. [41]	**H**	**H**	**L**	**H**	**H**	**High**
Mokhlesin et al. 42]	**L**	**Some concerns**	**L**	**L**	**L**	**Low**
Serel Arslan et al. [43]	**H**	**H**	**L**	**L**	**L**	**Some concerns**

Terminology used by author(s). *Notes:* RAND = Randomisation; DEV = Deviations from intended interventions; MIS_OUT = Missing outcome data; MEAS_OUT = Measurement of the outcome; SEL_REP = Selection of the reported result; OVERALL = Overall bias; H = High risk; L = Low risk.

## Data Availability

Dataset available on request from the authors.

## Abbreviations

AOT	Action Observation Training
babiEAT	baby intensive Early active Treatment
BPFAS	Behavioural Pediatrics Feeding Assessment Scale
CP	Cerebral Palsy
DSFS	Drooling Severity and Frequency Scale
FIPQ	Feeding Intervention Preferences Questionnaire
FOIS	Functional Oral Intake Scale
FOISi	Functional Oral Intake scale for Infants
FOMS	Feeding Oral Motor Scale
FS-IS	Feeding and Swallowing Impact Survey
FSIS	Feeding Swallowing Impact Scale
FuCT	Functional Chewing Training
GAS	Goal Attainment Scale
GMFM-88	Gross Motor Function Measure-88
IDDSI	International Dysphagia Diet Standardisation Initiative
ICF-CY	International Classification of Functioning-Children and Youth
KCPS	Karaduman Chewing Performance Scale
NCRT	Nutrition Related Caregiver Training
NDT-B	Neurodevelopmental Therapy Method-Bobath
OM	Oral Motor
OMAS	Oral Motor Assessment Scale
OMIS	Oral Motor Intervention Strategies
OMT	Oral Motor Therapy
OPD	Oropharyngeal Dysphagia
OSMS	Oral Sensorimotor Stimulation
Pedi-Eat	Pediatric Eating Assessment Tool,
PedsQL	Pediatric Quality of Life Inventory
PT	Physiotherapist
QoL	Quality of Life
SATCo	Segmental Assessment of Trunk Control
SLP	Speech-Language Pathologist
SLT	Speech and Language Therapist
SOMA	Schedule for Oral Motor Assessment
TIS	Trunk Impairment Scale
